# Virtual *In-Silico* Modeling Guided Catheter Ablation Predicts Effective Linear Ablation Lesion Set for Longstanding Persistent Atrial Fibrillation: Multicenter Prospective Randomized Study

**DOI:** 10.3389/fphys.2017.00792

**Published:** 2017-10-11

**Authors:** Jaemin Shim, Minki Hwang, Jun-Seop Song, Byounghyun Lim, Tae-Hoon Kim, Boyoung Joung, Sung-Hwan Kim, Yong-Seog Oh, Gi-Byung Nam, Young Keun On, Seil Oh, Young-Hoon Kim, Hui-Nam Pak

**Affiliations:** ^1^Cardiovascular Center, Korea University, Seoul, South Korea; ^2^Division of Cardiology, Yonsei University Health System, Seoul, South Korea; ^3^Division of Cardiology, Catholic University of Korea, Seoul, South Korea; ^4^Asan Medical Center, University of Ulsan, Seoul, South Korea; ^5^Samsung Medical Center, Sungkyunkwan University, Seoul, South Korea; ^6^Division of Cardiology, Seoul National University, Seoul, South Korea

**Keywords:** atrial fibrillation, catheter ablation, virtual ablation, *in-silico* modeling, recurrence

## Abstract

**Objective:** Radiofrequency catheter ablation for persistent atrial fibrillation (PeAF) still has a substantial recurrence rate. This study aims to investigate whether an AF ablation lesion set chosen using *in-silico* ablation (V-ABL) is clinically feasible and more effective than an empirically chosen ablation lesion set (Em-ABL) in patients with PeAF.

**Methods:** We prospectively included 108 patients with antiarrhythmic drug-resistant PeAF (77.8% men, age 60.8 ± 9.9 years), and randomly assigned them to the V-ABL (*n* = 53) and Em-ABL (*n* = 55) groups. Five different *in-silico* ablation lesion sets [1 pulmonary vein isolation (PVI), 3 linear ablations, and 1 electrogram-guided ablation] were compared using heart-CT integrated AF modeling. We evaluated the feasibility, safety, and efficacy of V-ABL compared with that of Em-ABL.

**Results:** The pre-procedural computing time for five different ablation strategies was 166 ± 11 min. In the Em-ABL group, the earliest terminating blinded *in-silico* lesion set matched with the Em-ABL lesion set in 21.8%. V-ABL was not inferior to Em-ABL in terms of procedure time (*p* = 0.403), ablation time (*p* = 0.510), and major complication rate (*p* = 0.900). During 12.6 ± 3.8 months of follow-up, the clinical recurrence rate was 14.0% in the V-ABL group and 18.9% in the Em-ABL group (*p* = 0.538). In Em-ABL group, clinical recurrence rate was significantly lower after PVI+posterior box+anterior linear ablation, which showed the most frequent termination during *in-silico* ablation (log-rank *p* = 0.027).

**Conclusions:** V-ABL was feasible in clinical practice, not inferior to Em-ABL, and predicts the most effective ablation lesion set in patients who underwent PeAF ablation.

## Introduction

The prevalence of AF increases with age, and ~20% of all ischemic strokes are associated with AF (Goldstein et al., 2011). It is predicted that the prevalence of AF would more than double by the year 2050 (Ball et al., 2013). Although radiofrequency catheter ablation (RFCA) has been established as an effective rhythm control strategy, it still has a substantial long-term recurrence rate, especially in patients with persistent AF (Dewire and Calkins, 2013). AF catheter ablation introduces the risk of collateral damages, and a long duration of ablation procedure is associated with poor clinical outcomes (Shim et al., 2013). Moreover, the ablation strategy is selected largely based on the physician's experience, which bears the possibility of outcome differences depending on the physician. On the other hand, computer simulation has been widely used in the study of the mechanisms of AF (Hwang et al., 2014; Trayanova, 2014). Recently, a number of attempts have been made to use computer simulation for studying the effects of catheter ablation (Zhao et al., 2015). We previously conducted a retrospective study and reported that the earliest virtual AF terminating lesion set chosen by using a computer was commonly identical to that selected by the physician who performed the ablations in patients with persistent AF (Hwang et al., 2014). Because the computing speed for complicated *in-silico* modeling of AF is one of the limitations for the application of computer simulation in clinical practice, we have shortened the computing time for AF modeling by about 80 times compared with conventional modeling (Hwang et al., 2015; Zhao et al., 2015). This study was a prospective randomized trial to examine the applicability, safety, and efficacy of computer simulation for catheter ablation of AF. The ablation strategy was selected on the basis of computer simulation for one group of patients, and according to the physician's experience for the other group. Then, we evaluated the feasibility of *in-silico* ablation (computing time), and compared procedure time, procedure related complication rate, long-term rhythm outcome of both groups, and the most effective lesion set in *in-silico* ablation and empirical ablation (Em-ABL).

## Methods

### Study design

**Figure 1:**
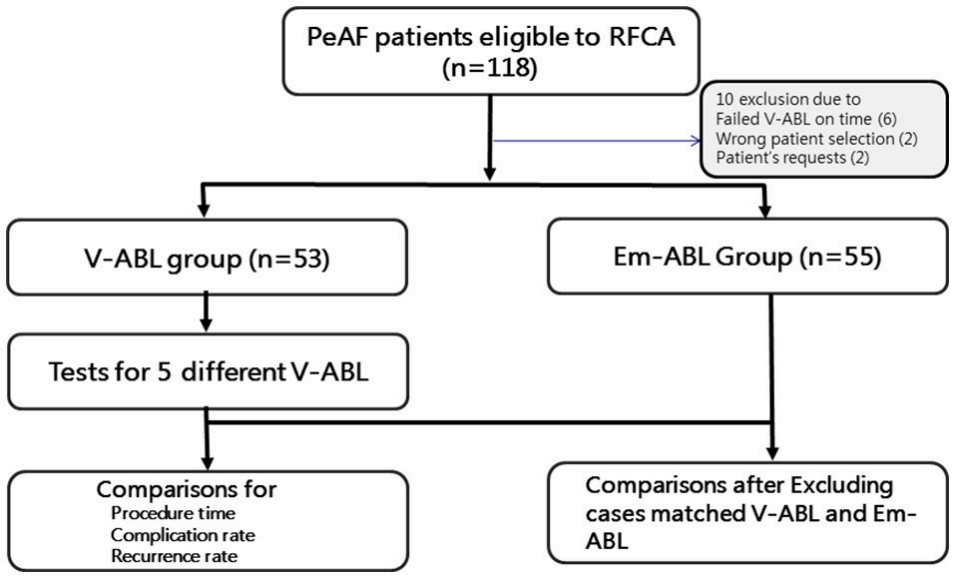
Study flow chart. The enrolled patients were randomly assigned to either the virtual ablation (V-ABL) group or the empirical ablation (Em-ABL) group. Virtual ablations were performed for all patients in both groups, by applying five strategies. For patients in the V-ABL group, the best virtual ablation strategy was applied in real ablation. For patients in the Em-ABL group, the ablation strategy selected by the physician based on experience was applied in real ablation. The ablation outcomes were compared between the V-ABL and Em-ABL groups.

The study protocol adhered to the Declaration of Helsinki and was approved by the institutional review board of the Yonsei University Health System (clinicaltrials.gov; NCT 02171364). The enrolled patients were randomly assigned to either the virtual ablation (V-ABL) group or the Em-ABL group (Figure 1). For all patients in both groups, virtual ablations were performed by applying the following 5 strategies: (i) circumferential pulmonary vein isolation (CPVI), (ii) CPVI + posterior box lesion, (iii) CPVI + posterior box lesion + anterior line, (iv) CPVI + roof line + left lateral isthmus line, and (v) CPVI + complex fractionated atrial electrogram (CFAE)-guided ablation (Hwang et al., 2014; Song et al., 2016). For patients in the V-ABL group, the best virtual ablation strategy was applied in real ablation. The virtual ablation strategy that resulted in the earliest termination of AF after *in-silico* ablation was determined as the best *in-silico* ablation lesion set among the five ablation strategies. For patients in the Em-ABL group, the ablation strategy selected by the physician based on experience was applied in real ablation. The ablation outcomes were compared between the V-ABL and Em-ABL groups.

### Study population

In this study, we enrolled 118 patients with persistent AF (PeAF) who were eligible for RFCA. All patients provided written informed consent. The inclusion criteria were (i) age 19 years or older, (ii) antiarrhythmic drug resistance, and (iii) availability of three-dimensional (3D) computed tomography (CT) or magnetic resonance imaging (MRI) scans of the left atrium (LA). The exclusion criteria were (i) presence of significant structural heart disease, (ii) valvular AF, (iii) previous ablation or cardiac surgery, and (iv) presence of a cardiac implantable electronic device.

After receiving the patients' consent, DICOM files of heart CT were sent to the core laboratory a night before or in the early morning of the real AF ablation procedure. Patient randomization, *in-silico* AF modeling, and virtual AF ablation were done at the core laboratory during work hours (9.a.m.–6 p.m.). Among 118 patients, four were excluded owing to incorrect patient selection or patient request. Additional six patients were excluded because of failed *in-silico* AF ablation before the real ablation procedure, due to DICOM file error (*n* = 2) and communication error (*n* = 4). Finally, 108 patients (53 in the V-ABL group and 55 in the Em-ABL group) were enrolled in this study (Figure 1). The patient characteristics are shown in Table 1.

**Table 1:** Patient characteristics.

	**Overall (*N* = 108)**	**V-ABL (*N* = 53)**	**Em-ABL (*N* = 55)**	***p*-Value**
Age (years)	60.8 ± 9.6	59.7 ± 10.1	61.9 ± 9.6	0.240
Male (%)	76.9	75.5	78.2	0.821
Longstanding persistent AF (%)	77.8	83.0	72.7	0.249
CHA2DS2-VASc score	1.97 ± 1.86	1.85 ± 1.65	2.09 ± 1.86	0.475
Heart failure (%)	12.0	9.4	14.5	0.557
Hypertension (%)	54.6	52.8	56.4	0.847
Age > 75 years (%)	9.3	3.8	14.5	0.094
Age 65–74 years (%)	25.0	28.3	21.8	0.508
Diabetes (%)	18.5	17.0	20.0	0.806
Previous stroke (%)	28.7	28.3	29.1	1
Previous TIA (%)	1.9	3.8	0	0.238
Vascular disease (%)	13.0	9.4	16.4	0.392
**ECHOCARDIOGRAPHIC FINDINGS**				
LA dimension (mm)	45.1 ± 4.4	46.1 ± 7.6	44.0 ± 4.4	0.086
EF (%)	59.3 ± 9.7	57.8 ± 7.8	60.7 ± 9.7	0.092
E/Em	10.2 ± 4.7	9.6 ± 3.0	10.7 ± 4.7	0.139

*TIA, transient ischemic attack; LA, left atrium; EF, ejection fraction; E/Em, the ratio of early transmitral flow velocity (E) to early mitral annular velocity (Em)*.

### *In-silico* simulation of AF

Atrial geometries were reconstructed from the 3D CT merged NavX data (NavX; St. Jude Medical Inc., Minnetonka, MN, USA) defining the surface of the atrium. A triangular mesh was generated on the surface of the 3D atrial geometry. The final number of grid elements was between 400,000 and 500,000. The LA appendage and myocardial sleeves were included in the mesh. For the simulation of cardiac wave propagation in the atrial wall, the following reaction-diffusion equation was solved numerically (Zozor et al., 2003):

(1) $$\begin{array}{r} \frac{\partial V_{m}}{\partial t}=\frac{1}{\beta C_{m}}\left\{\nabla \cdot D\nabla V_{m}-\beta \left(I_{ion}+I_{s}\right)\right\}, \end{array}$$

where *Vm* is the membrane potential (Unit: volt); β is the membrane surface-to-volume ratio (Unit: meter^−1^); *C*_*m*_ is the membrane capacitance per unit area (Unit: farad/meter^2^); D is conductivity tensor (Unit: siemens/meter); and *I*_*ion*_ and *I*_*s*_ are the ion current and stimulation current, respectively (Unit: ampere/meter^2^).

For the calculation of ionic currents, a mathematical model of human atrial action potential proposed by Courtemanche et al. (1998) was used. Electrical stimulation was applied at the location of Bachmann's bundle, and reentry was initiated by rapid pacing: a total of 24 pacings with pacing cycle lengths of 200, 190, and 180 ms. To induce self-sustained fibrillation, I_to_, I_CaL_, and I_Kur_ were reduced by 80, 40, and 50%, respectively, and I_K1_ was increased by 50% (Li et al., 2016). We chose a conduction velocity of 0.4 m/s based on real human patient data (Yonsei AF ablation cohort data; n = 1,980; mean CV = 0.43 ± 0.24 m/s) (Park et al., 2014) and previous simulation studies (Hwang et al., 2014; Li et al., 2016).

### Virtual AF ablation

**Figure 2:**
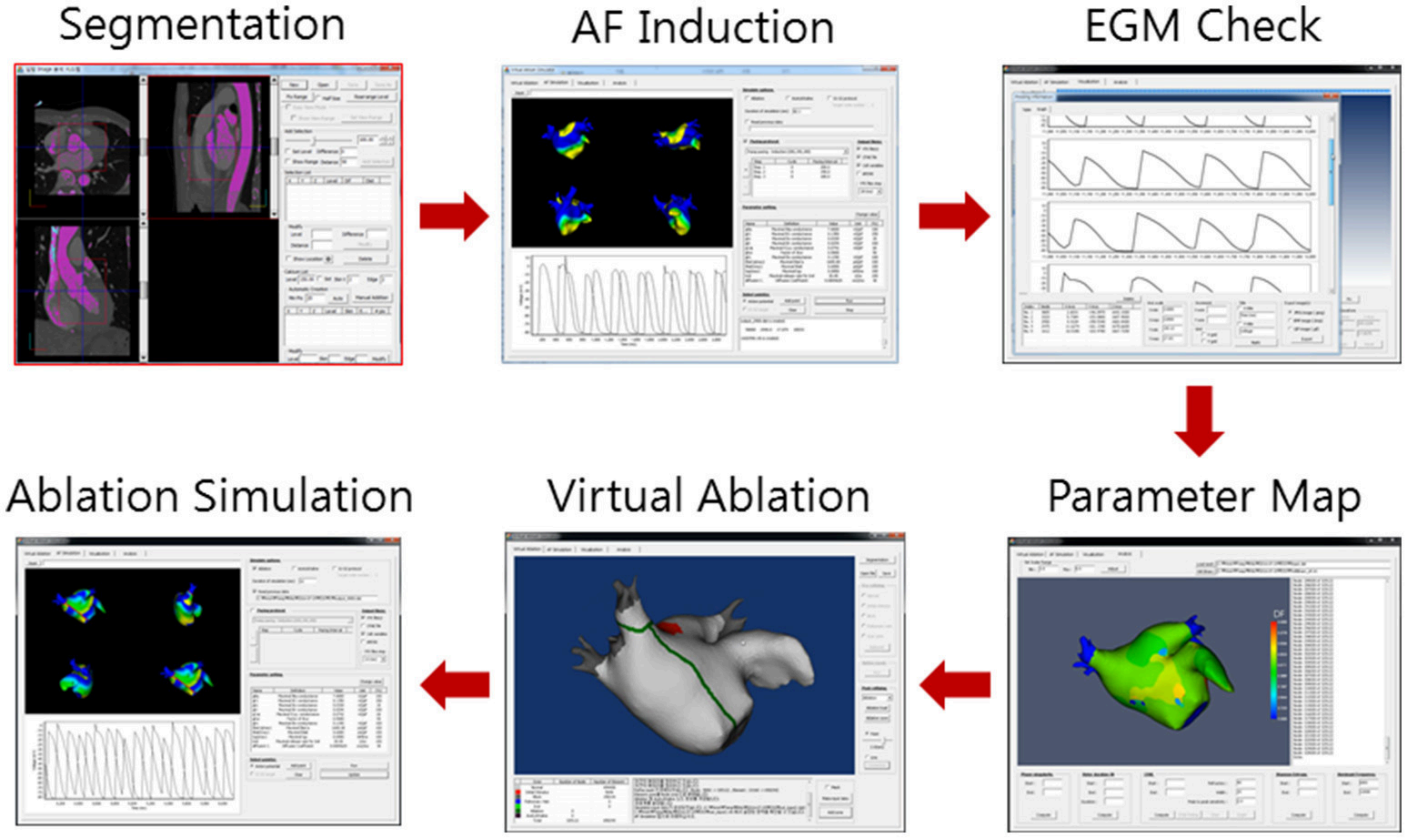
Virtual ablation procedure. Computed tomography images are segmented to construct three-dimensional atrial geometry. Virtual atrial fibrillation (AF) is induced. The maps of cardiac wave dynamics parameters such as complex fractionated atrial electrogram (CFAE) are generated. Virtual ablation is performed on the atrial geometry. AF simulation is resumed. The cardiac wave propagation pattern is observed.

Virtual ablation was conducted for all patients in both the V-ABL and Em-ABL groups. We developed a graphical user interface software with which the user can perform virtual ablation by mouse-clicking on the atrial geometry (CUVIA, Model: SH01, ver. 1.0; Laonmed Inc., Seoul, Korea; Figure 2). At the point where the mouse is clicked, a 2-mm-diameter circular region is virtually ablated, mimicking real ablation with a radiofrequency catheter. In the ablated region, no electrical flux condition was applied. The pattern of ablation was completed by serial mouse-clickings, similarly to real catheter ablation. For CFAE-guided ablations (Nademanee et al., 2004), the areas with CFAE cycle length < 120 ms were ablated as long as the total ablated area is < 5% of the whole atrial area. CFAE was observed in particular region in structurally homogenous simulation due to the three-dimensional geometrical variation. The area where the mitral annulus was located was also considered a non-conductive area. Virtual ablation was applied at 4 s after the end of pacings, and the simulation was run until 25 s from the start of the simulation. When all points in the atrial wall were repolarized by > 90%, the fibrillation was considered terminated. The duration until the termination of fibrillation was recorded for each ablation strategy for each patient. NavX potential map was not used in the virtual ablation. The physicians, however, utilized the endocardial potential map data in their decision makings. Fiber orientation was not considered in the simulation model, and conductivity was isotropic even though three-dimensional geometrical shape may have affected conductivity.

### Real AF ablation

The electrophysiological mapping method and the RFCA technique were as described previously (Mun et al., 2012). Briefly, we used an open irrigated-tip catheter (Celsius [Johnson & Johnson Inc., Diamond Bar, CA, USA] or Coolflex [St. Jude Medical]; 30–35 W, 47°C) to deliver radiofrequency energy for ablation under 3D electroanatomical mapping (NavX, St. Jude Medical; CARTO3, Johnson & Johnson) merged with 3D spiral CT. The procedure was completed when there was no immediate recurrence of AF after cardioversion with isoproterenol infusion (5 μg/min). All RFCA procedures were performed by an operator with > 10 years of experience.

### Post-ablation management and follow-up

As per RFCA procedure protocols, antiarrhythmic drugs were discontinued in all patients. The patients visited the outpatient clinic at 1, 3, 6, and 12 months after RFCA, or whenever symptoms occurred. All patients underwent electrocardiography at each visit, and 24-h Holter recording was done at 3, 6, and 12 months, according to the 2012 Heart Rhythm Society/European Heart Rhythm Association/European Cardiac Arrhythmia Society Expert Consensus Statement (Calkins et al., 2012). However, whenever patients reported palpitations, Holter monitor or event monitor recordings were obtained and evaluated to check for arrhythmia recurrence. We defined recurrence of AF as any episode of AF or atrial tachycardia lasting for at least 30 s. Any electrocardiography documentation of AF recurrence after 3 months of the blanking period was diagnosed as clinical recurrence.

### Statistical analysis

Because the trial was conducted as a pilot study, the sample size was driven by the computation time and feasibility of recruiting eligible patients, and enrollment was open for 12 months. Results are expressed as mean values ± standard deviation for continuous variables, and absolute number and percentages for categorical variables. Continuous variables were compared by using Student's *t*-test, and categorical variables were compared by using either the chi-square test or Fisher's exact test as appropriate. The primary end point of the study was freedom from any atrial arrhythmias during follow-up after a 3-month blanking period. The time to recurrence and arrhythmia-free survival were assessed by using Kaplan-Meier analysis, and differences were calculated by using the log-rank test. A *p*-value of < 0.05 was considered statistically significant. All statistical analyses were performed by using SPSS version 18.0 software (SPSS Inc., Chicago, IL, USA).

## Results

### Patient characteristics

The patients' characteristics are summarized in Table 1. The mean age was 60.8 ± 9.6 years, and 76.9% were male patients. Among 108 patients with PeAF, 77.8% had longstanding PeAF (lasting for >1 year). More than 28% of patients had a history of stroke or transient ischemic attack; the mean left atrial dimension was 45.1 ± 4.4 mm and the mean ejection fraction was 59.3 ± 9.7%. Randomization was done well, and there was no statistically significant difference between the V-ABL and Em-ABL groups.

### *In-silico* ablation results

*In-silico* AF ablations were done in all included patients; however, the Em-ABL group was blinded to the *in-silico* ablation results. The pre-procedural computing time for the five virtual ablation strategies described in the Methods section was 166 ± 11 min. Heart CT segmentation with a semi-automatic method and five different *in-silico* ablation lesion settings with the manual method took about 1 h. Another 1 h was taken for computing the five different *in-silico* ablation protocols simultaneously, and additional 30 min was required for the calculation of the CFAE area.

Table 2 summarizes the outcome of *in-silico* AF ablation. After virtual ablation, we waited for 25 s, measuring the termination time. Among the five *in-silico* ablation strategies, [CPVI+Posterior box+Anterior linear ablation] showed the highest AF termination rate (81.5%, 88 of 108) and shortest time to AF termination (16.792 ± 5.672 ms). The AF termination rates within 25 s were 73.1% after [CPVI+Roof line+Left lateral isthmus] *in-silico* ablation, 28.7% after [CPVI+Posterior box] after *in-silico* ablation, 11.1% after [CPVI only], and 8.3% after [CPVI+CFAE] virtual ablation. Compared with virtual [CPVI only], virtual [CPVI+Posterior box+Anterior line] (*p* < 0.001) and virtual [CPVI+Roof line+Left lateral isthmus line] (*p* < 0.001) showed significantly higher virtual AF termination rates in both the V-ABL and Em-ABL groups. There was no significant difference in the *in-silico* ablation outcome between the V-ABL group and the Em-ABL group.

**Table 2:** Virtual ablation outcome.

	**Overall (*N* = 108)**	**V-ABL (*N* = 53)**	**Em-ABL (*N* = 55)**	***p*-Value**
Conduction velocity (m/s)	0.41 ± 0.11	0.40 ± 0.07	0.41 ± 0.14	0.615
APD_90_ (ms)	213 ± 2	213 ± 2	213 ± 3	0.618
**AF TERMINATION RATE (%)**				
CPVI	11.1 (12/108)	9.4 (5/53)	12.7 (7/55)	0.761
CPVI + PostBox	28.7 (31/108)^*^	34.0 (18/53)^†^	23.6 (13/55)	0.289
CPVI + PostBox + AL	81.5 (88/108)^*^	83.0 (44/53)^†^	80.0 (44/55)^‡^	0.806
CPVI + RL + LLI	73.1 (79/108)^*^	75.5 (40/53)^†^	70.9 (39/55)^‡^	0.667
CPVI + CFAE	8.3 (9/108)	7.5 (4/53)	9.1 (5/55)	1.000
**TIME TO AF TERMINATION (MS)**				
CPVI	23914 ± 3466	24017 ± 3354	23815 ± 3599	0.763
CPVI + PostBox	21893 ± 5471	21463 ± 5719	22307 ± 5240	0.426
CPVI + PostBox + AL	16792 ± 5672	16478 ± 5750	17094 ± 5633	0.575
CPVI + RL + LLI	17701 ± 5770	17199 ± 5949	18185 ± 5604	0.378
CPVI + CFAE	24170 ± 3041	24319 ± 2686	24018 ± 3385	0.619

* p < 0.001,

† p < 0.001,

‡ *p < 0.001 compared with the CPVI of each group*.

*APD_90_, action potential duration of 90% repolarization; CPVI, circumferential pulmonary vein isolation; PostBox, posterior box isolation; AL, anterior line; RL, roof line; LLI, left lateral isthmus line; CFAE, complex fractionated atrial electrogram guided ablation*.

### Comparison of V-ABL and Em-ABL

In the real ablation procedure, CPVI only was more commonly chosen in the Em-ABL group (30.9%) than in the V-ABL group (1.9%, *p* < 0.001; Table 3). Among patients in the V-ABL group, [CPVI+Roof+Left lateral isthmus] ablation was most commonly chosen (43.4%), although *in-silico* AF termination rate was highest in [CPVI+Posterior box+Anterior line] (Table 2). This was because, when AF was terminated in both lesion sets (32 cases), we chose the earliest terminating virtual lesion set (16 in [CPVI+Roof+Left lateral isthmus] and 11 in [CPVI+Posterior box+Anterior line]). In the Em-ABL group, [CPVI+Posterior box+Anterior line] was selected most often (36.4%), followed by [CPVI only] (30.9%; *p* = 0.687) (Table 3). Among patients in the Em-ABL group, the earliest terminating *in-silico* lesion set, which was blinded to the operator, was identical to the empirically chosen real ablation lesion set in 21.8% (12 of 55).

**Table 3:** Clinical outcome.

	**Overall (*N* = 108)**	**V-ABL (*N* = 53)**	**Em-ABL (*N* = 55)**	***p*-Value**
**PROCEDURAL LESION SET (%)**				
CPVI	16.7 (18/108)	1.9 (1/53)	30.9 (17/55)	<0.001
CPVI + PostBox	6.5 (7/108)	11.3 (6/53)	1.8 (1/55)	0.058
CPVI + PostBox + AL	38.0 (41/108)	39.6 (21/53)	36.4 (20/55)	0.843
CPVI + RL + LLI	33.3 (36/108)	43.4 (23/53)	23.6 (13/55)	0.041
CPVI + CFAE	5.6 (6/108)	3.8 (2/53)	7.1 (4/56)	0.679
Procedure time (min)	263.5 ± 88.5	256.2 ± 69.0	271.5 ± 104.7	0.403
Ablation time (min)	5121.9 ± 2574.6	4954.7 ± 2804.0	5272.8 ± 2368.2	0.510
Fluoroscopic time (min)	57 ± 30	59 ± 31	55 ± 30	0.523
Complication rate (%)	4.2	4.4	4.0	0.900
AAD utilization rate (%)	42.6	49.1	36.4	0.320
Early recurrence (%)	30.2	33.3	27.3	0.531
Clinical recurrence (%)	16.0	14.0	18.9	0.538

*CPVI, circumferential pulmonary vein isolation; PostBox, posterior box isolation; AL, anterior line; RL, roof line; LLI, left lateral isthmus line; CFAE, complex fractionated atrial electrogram guided ablation; AAD, antiarrhythmic drug*.

The procedure-related acute outcomes are summarized in Table 3. There was no significant difference in procedure time (*p* = 0.289), ablation time (*p* = 0.988), complication rates (*p* = 0.359), and early recurrence rate within 3 months (*p* = 1.000) between the V-ABL group and the Em-ABL group.

### Comparison of rhythm outcomes

**Figure 3:**
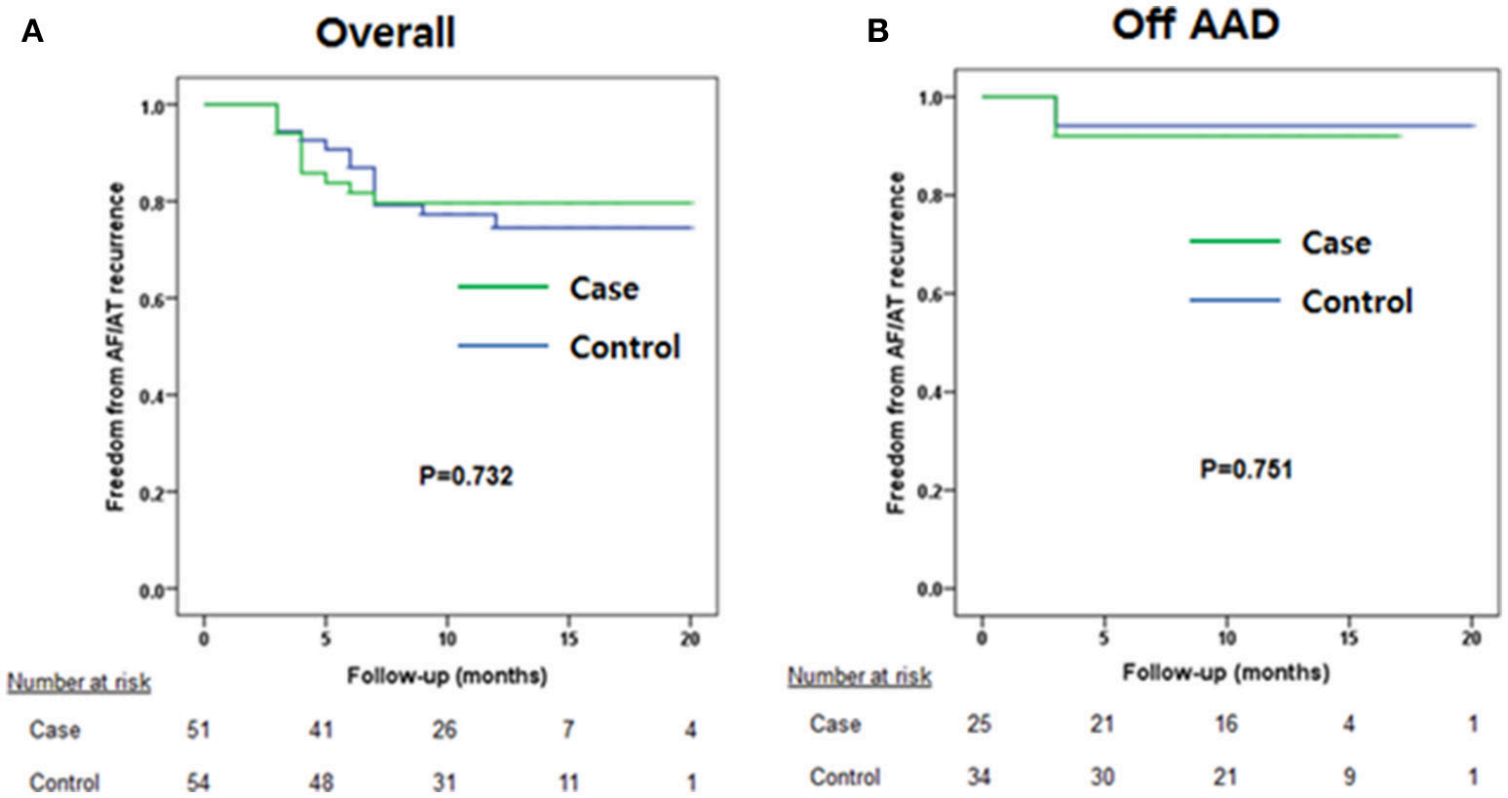
Kaplan-Meier curves. **(A)** Overall patients. **(B)** Patients maintaining antiarrhythmic drug use were excluded.

During 12.6 ± 3.8 months of follow-up, the clinical recurrence rates were 14.0% in the V-ABL group and 20.9% in the Em-ABL group (*p* = 0.411). The maintenance rates of antiarrhythmic drugs were 49.1% in the V-ABL group and 39.5% in the Em-ABL group (*p* = 0.840). Kaplan-Meier analyses showed consistent findings in the overall patients (log-rank *p* = 0.732; Figure 3A) and after excluding patients who maintained antiarrhythmic drugs (log-rank *p* = 0.751; Figure 3C).

**Figure 4:**
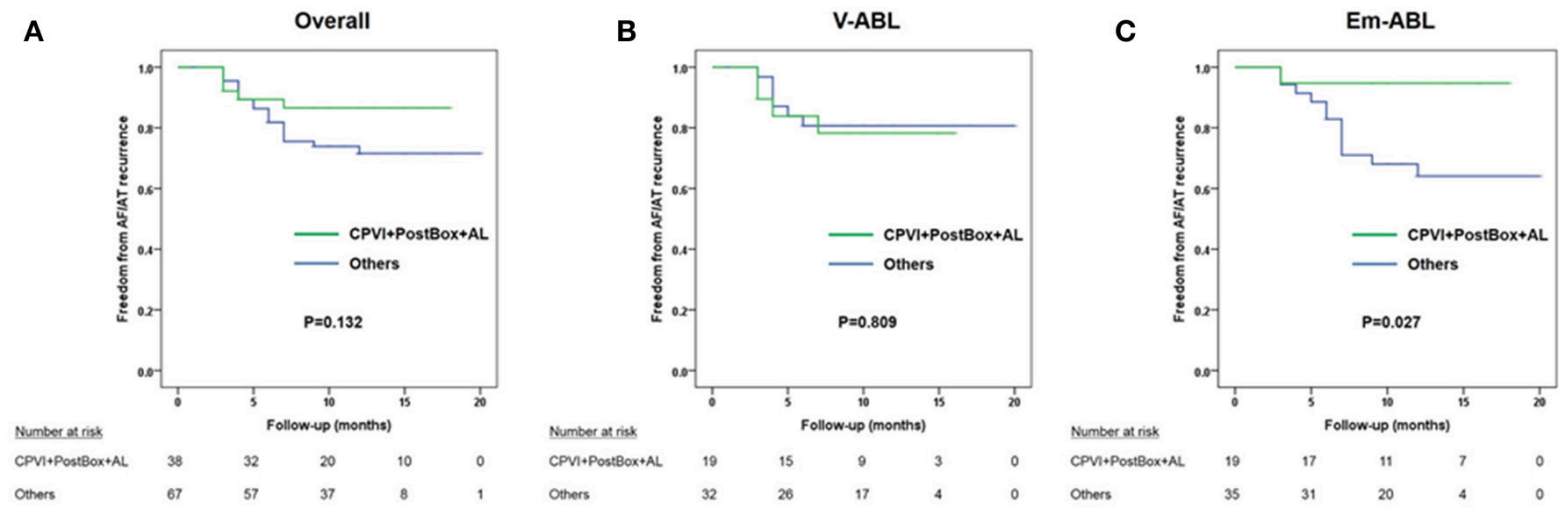
Kaplan-Meier curves comparing the clinical recurrence rate of patients who underwent circumferential pulmonary vein isolation (CPVI) + posterior box lesion + anterior line with that of the other patients. **(A)** Overall patients. **(B)** V-ABL group. **(C)** Em-ABL group.

However, when we compared the clinical recurrence rate according to the ablation lesion set, the clinical recurrence rates were significantly lower in patients in the Em-ABL group who underwent [CPVI+Posterior box+Anterior line] ablation, which showed the most frequent termination and shortest AF maintenance duration in virtual ablation (log-rank *p* = 0.027; Figure 4).

## Discussion

### Main findings

In this prospective randomized trial, we investigated the feasibility of using a virtual ablation system in clinical practice and compared the clinical outcome of simulation-guided ablation (V-ABL) with empirical ablation (Em-ABL) in patients with PeAF. Patient-specific atrial geometry was used for finite element modeling, and five different virtual ablation patterns were tested for each patient. This is the first randomized study to validate the clinical outcomes of simulation-guided ablation, demonstrating the potential of *in-silico* computer models for planning individualized ablation strategies in clinical practice. We demonstrated that the most effective *in-silico* AF ablation lesion set showed the best clinical outcome in the Em-ABL group. The overall safety and efficacy of simulation-guided intervention was not inferior to empirical ablation of PeAF.

### Unmet clinical needs in PeAF ablation

Although pulmonary vein isolation is the cornerstone of catheter ablation for AF and effective in patients with paroxysmal AF, this was found to be insufficient to treat longstanding persistent AF. Even in the STAR AF II trial (Verma et al., 2015), which advocated CPVI alone for PeAF, a 41% recurrence rate within 18 months of CPVI procedure was not good enough for clinical practice. However, there is no consensus on further ablation after CPVI in PeAF (Verma et al., 2015). Linear ablation modifies the atrial substrates and compartmentalizes the atrium into smaller regions to reduce critical mass and AF maintenance (Pak et al., 2011). The question on what is the best linear lesion set for individual patients remains unanswered. CFAE ablation has been reported to be effective for substrate modification in PeAF by some investigators (Nademanee et al., 2004; Verma et al., 2010); however, this positive outcome was not reproduced in other investigations (Oral et al., 2008). Other various ablation strategies targeting rotor (Narayan et al., 2012), driver (Haissaguerre et al., 2014), dominant frequency (Atienza et al., 2014), or Shannon entropy (Ganesan et al., 2013) remain to be validated in patients with PeAF. There are potential explanations for the lack of a “one-fits-all ablation protocol.” First, PeAF is a progressive disease with various stages, and its nature is affected by the anatomy, histology, electrophysiology, and hemodynamic loading of individual patients. Second, the AF ablation procedure is operator dependent and the outcome is affected by the ablation lesion set; catheter stability; or ablation power, duration, and contact force. Third, it is not possible to test three different ablation designs in a patient because radiofrequency ablation causes irreversible tissue change. Therefore, *in-silico* AF ablation might be useful to test several different ablation lesion sets in a personalized modeling approach reflecting anatomy, histology, electrophysiology, and even genetic characteristics (Hwang et al., 2014).

### Roles and limitations of *in-silico* AF ablation

The advantage of computer simulation modeling is the potential for direct comparison of different ablation strategies for individual pre-procedural planning. As a result, modeling is emerging as a complementary approach to animal experiments and clinical trials in investigating more effective treatment (Winslow et al., 2012). Previously, Ruchat et al. (2007) compared the success rates in converting AF to sinus rhythm for 19 different linear ablation patterns by using a single identical human atrial model. There have been six AF simulation studies of catheter ablation thus far (Jacquemet, 2016), and we previously reported on multiple virtual ablation protocols in 20 different personalized human atrial models (Hwang et al., 2014). In this study, we conducted *in-silico* ablations prospectively in 108 personalized LA models reflecting individual anatomy; however, it was also a single-layer homogeneous modeling approach without integrating local histology. The main reason for our use of over-simplified simulation modeling was to reduce the computing time. Unless AF simulation and virtual intervention can generate the result within a few hours, it is not acceptable for clinical application during intervention. Although we already made the computing time 80 times faster by using a graphics processing unit system, further innovation is still needed for sophisticated realistic AF modeling reflecting detailed histology and electrophysiology.

Another discrepancy of *in-silico* AF ablation and real catheter ablation is completeness and maintenance of conduction block after linear ablation. Although [CPVI+Posterior box+Anterior linear ablation] showed the highest antifibrillatory effect in both virtual ablation and Em-ABL, it was difficult to achieve complete and permanent transmural conduction block by using endocardial radiofrequency energy delivery in the clinical setting with a beating heart and respiratory motion. A non-transmural lesion or ablation gap provides one of the main reasons for AF recurrence after an ablation procedure, discordant to *in-silico* AF ablation.

### Study limitations

There are limitations in our simulation modeling. The mechanism of AF in this particular computer model is based exclusively on multiple wavelet theories (Moe et al., 1964), and other well-described mechanisms like rotors can be masked in this model. Although we used patient-specific atrial geometry for the AF ablation simulation, individual electrophysiological and structural characteristics such as regional differences in action potential morphology, and fiber orientation were not included. Clinical studies showed that atrial wall thickness affected AF wave dynamics (Song et al., 2017), but the wall thickness was not considered in the present model. We also did not model the right atrium, because AF drivers have been known to exist mostly in the LA in humans (Sanders et al., 2005). We assume that the predictability of AF intervention by using simulation will be improved remarkably by integrating high-resolution cardiac imaging modalities reflecting the remodeled scar area, pathological electro-anatomical information acquired during procedure, and more realistic multilayer bi-atrial modeling study. Although our modeling protocol for virtual AF ablation may not reflect the mechanism of AF ablation precisely (whether virtual ablation stops an ongoing AF or whether it hinders the onset of new episode of AF, ectopic centers, rotor, or micro-reentry, etc.), it exhibits the antiarrhythmic effects of appropriate critical mass reduction, which is the main antiarrhythmic mechanism of linear ablation of AF or a surgical maze procedure. While CV may also change AF wave dynamics, we used a fixed CV based on real human patient data (Yonsei AF ablation cohort) (Park et al., 2014) and previous simulation studies (Hwang et al., 2014; Li et al., 2016). Because we tested five different virtual ablation protocols in every patient's LA modeling at the same CV condition, it did not affect the mechanism of critical mass reduction. For the model to show superiority to empirical ablation, individual patient's structural heterogeneity such as scar/fibrosis, fiber orientation, and wall thickness variation should be included in the model.

## Conclusion

This is the first randomized multicenter study to demonstrate that simulation-guided ablation by using personalized models of LA was feasible, and the clinical outcome was not inferior to that of empirical ablation. Although there are limitations in applying virtual AF ablation to a personalized ablation strategy, our results showed that the virtual AF ablation system is capable of identifying the most effective ablation lesion set for individual patients. Advances in modeling technology might provide useful clinical insights for planning therapeutic interventions in the near future.

## Author contributions

Conception and design of the study: HP. Acquisition of data: JS, TK, BJ, SK, YSO, GN, YKO, SO, YK, and HP. Analysis of data: JS, MH, JSS, and BL. Interpretation of data: JS and HP. Drafting the work: JS and MH. All authors contributed in revising the work, approved the final version to be published, and agreed to be accountable for all aspects of the work.

### Conflict of interest statement

The authors declare that the research was conducted in the absence of any commercial or financial relationships that could be construed as a potential conflict of interest.
